# Demonstrating real-time and low-latency quantum error correction with superconducting qubits

**DOI:** 10.1038/s41467-026-73331-6

**Published:** 2026-06-01

**Authors:** Laura Caune, Luka Skoric, Nick S. Blunt, Archibald Ruban, Jimmy McDaniel, Joseph A. Valery, Andrew D. Patterson, Alexander V. Gramolin, Joonas Majaniemi, Kenton M. Barnes, Tomasz Bialas, Okan Buğdaycı, Ophelia Crawford, György P. Gehér, Hari Krovi, Elisha Matekole, Canberk Topal, Stefano Poletto, Michael Bryant, Kalan Snyder, Neil I. Gillespie, Glenn Jones, Kauser Johar, Earl T. Campbell, Alexander D. Hill

**Affiliations:** 1 Riverlane, Cambridge, UK; 2 Rigetti Computing, Berkeley, CA USA; 3 Rigetti UK Limited, London, UK; 4 Riverlane, Cambridge, MA USA; 5 School of Mathematical and Physical Sciences, Sheffield, UK

**Keywords:** Quantum information, Qubits

## Abstract

Quantum error correction will be essential for quantum computers to realise their full potential. As quantum computers advance towards demonstrating a universal fault-tolerant logical gate set, implementing scalable and low-latency real-time decoding will be crucial to avoid an exponential slowdown and maintain a fast logical clock rate. Here, we demonstrate low-latency feedback with a scalable FPGA decoder integrated into the control system of a superconducting quantum processor. We perform an 8-qubit stability experiment with up to 25 decoding rounds and a sub-microsecond mean decoding time per round, providing strong evidence that the backlog problem will be avoided when the decoder is operated as a streaming decoder on a superconducting hardware with the strictest speed requirements. We observe logical error suppression as the number of decoding rounds is increased. We also implement and time a fast-feedback experiment demonstrating a decoding response time of 9.6 *μ*s for a total of 9 measurement rounds.

## Abstract (web summary)

The authors report real-time quantum error correction on Rigetti’s Ankaa-2 by integrating a scalable FPGA decoder into the control stack achieving sub-1 *μ*s mean decoding per round and 9.6 us feedback latency. This work provides a concrete advancement on scalable fault-tolerant computing

## Introduction

Quantum computers have the potential to perform computations that are beyond the capabilities of classical computers^1,2^. However, various quantum algorithms that offer an advantage over classical algorithms will require hundreds of qubits and over a billion operations^3–6^, which are prone to errors. To combat the noise and unlock the potential of such algorithms, quantum error correction (QEC) will be necessary. QEC encodes the information we want to protect by distributing it across multiple qubits and repeatedly generating data characterising quantum errors^7–9^. Classical algorithms, known as decoders, use this data to identify errors that occurred during quantum computation. At low enough physical error rates, QEC suppresses errors when executing quantum algorithms, offering a path towards practical quantum computation. However, QEC codes alone cannot implement a universal and transversal gate set^10^. Leading proposals for implementing non-Clifford gates require logical branching – a logical operation conditional on a corrected observable, which is computed by combining the measured value of the observable and the logical correction returned by the decoder. For example, on the surface code^11–13^, a logical *T* gate can be implemented via teleportation and magic state injection^14–16^, which requires a conditional logical *S* gate. Since logical branching happens during circuit execution and depends on the decoding result, this places throughput and latency requirements on the decoding process.

The rate at which the decoder processes data (the throughput) needs to be greater than the rate at which data is generated, which on superconducting devices can be as fast as one data extraction round per 1 *μ*s^17–20^. This is necessary to avoid the backlog problem^7^, i.e., an exponential slowdown of the quantum computation due to a growing backlog of data at each decoding iteration. High throughput can be achieved both with fast decoders and by parallelizing the decoding workload over many decoder threads^21–25^. However, parallelization strategies will be limited by^21^ a second key parameter: the full decoding response time, which is the time between the final data extraction round and the application of a logical conditional gate. This response time includes the decoding time as well as the communication and control latency times to send the data to and from the decoder. This parameter can be a significant speed bottleneck for operations involving logical branching such as logical non-Clifford gates on surface codes^14–16^. As a result, it directly affects the logical clock rate (i.e., the inverse of the time required to perform a single logical non-Clifford gate), another QEC metric that determines the execution speed of fault-tolerant algorithms. Reducing both the decoding time and the communication and control latency to minimize the full decoding response time is key to ensure that complex quantum algorithms are executed within reasonable times. For example, when estimating that 2048-bit RSA integers can be factored in 8 h using 20 million noisy superconducting qubits, the authors assume a full decoding response time within 10 μs ^3^. To achieve decoding response time fast enough to support meaningful logical branching, decoding must occur on a time scale related to the duration of a QEC round (see Results subsection Control and communication latencies).

To summarize, a practical real-time decoding system must meet key requirements—to avoid the backlog problem and to support logical branching with low-latency decoding response times, all while being scalable. Table 1 highlights the different aspects of real-time decoding demonstrated in previous work^25,26^ and this work. Reference ^25^ experimentally demonstrates that by parallelizing their scalable, streaming decoder, no backlog of data is accumulated even with up to 10^6^ QEC rounds, but with decoding system latencies far exceeding QEC round time scales and without demonstration of logical branching. Ref. ^26^ operates their decoder in a streaming fashion and implements a logical branching experiment with low latencies, however, with an unscalable lookup table decoder and with millisecond time scales. Lookup table decoders reduced to if statements in the system have also been used in other previous real-time decoding experiments^27–29^.

**Table 1:** Summary of real-time decoding capabilities studied in this and previous work

Real-time decoding capability	This work (at *μ* s scale)	Ref. ^25^ (at *μ* s scale)	Ref. ^26^ (at *m*s scale)
High-throughput streaming decoding		*✓*	*✓*
Low-latency full decoding response time	*✓*		*✓*
Demonstrates logical branching	*✓*		*✓*
Scalable decoder	*✓*	*✓*	

A real-time decoding system for practical fault-tolerant quantum computation must demonstrate key capabilities: high-throughput streaming decoding, low-latency full decoding response time, and support for logical branching, all while being scalable. While real-time decoding has been demonstrated previously, prior work has shown different individual aspects of these necessary functionalities. In this table, we summarize the real-time decoding aspects and scalability of the decoder studied in this work as well as prior work on decoding in real time: a *d* = 5 surface code memory experiment with superconducting qubits^25^ using a scalable CPU correlated parallel blossom decoder and a [[7,1,3]] color code memory experiment on a trapped-ion quantum computer^26^ using a lookup table decoder. In this work, we focus on using a scalable FPGA decoder integrated in the control system of the Ankaa-2 QPU control system, demonstrating a logical branching experiment with fast full decoding response times and high throughput, while not implementing streaming decoding.

**Fig. 1:**
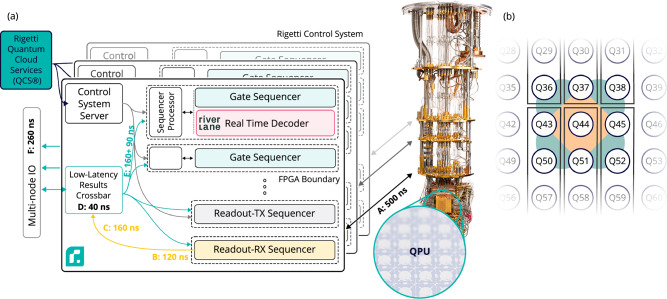
Integration of a hardware decoder into a control system. **a** Sketch of the Ankaa-2 quantum processing unit (QPU) and control system architecture with the integrated FPGA decoder. Inter-unit latencies are denoted with worst-case values where measured. A (in black): Delay between readout transmit and readout receive (approx. 500 ns). This delay contributes to overall latency but not to inter-shot or inter-readout delay; B (in yellow): Time required for readout receiver to generate a classified result, convert to intra-unit message, and serialize to the low-latency results crossbar (120 ns); C (in yellow): Time required for transmission from readout receiver to low-latency results crossbar (120-160 ns); D (in black): Time required to handle result message and prepare for broadcast (40 ns); E (in teal): Time required to transmit from low-latency results crossbar to internal controller option cards (120-160 ns), with additional time for readout-to-sequencer logic to send classified results to sequencers, within each card (40-90 ns); F (in black): Inter-node delay time for broadcasting between control system chassis (240-260 ns). **b** Mapping of Ankaa-2 sublattice to readout units. Each on-chip multiplexed readout feedline is associated with separate readout-RX and readout-TX gate cards. Because of where these qubits are located on their respective readout feedlines, the sublattice [Q36, Q37, Q38, Q43, Q44, Q45, Q50, Q51, Q52] corresponding to the stability experiment (shown in background and details in Results subsection Stability experiment on a surface code) falls across six different readout TX/RX hardware regions, marked with grey rectangles.

In this work, we demonstrate a scalable, high-throughput, low-latency decoding system supporting logical branching. We present a real-time decoded QEC experiment on 8 qubits with logical branching that measures the full decoding response time to be 9.6 μs, including 6.5 μs decoding time and 3.1 μs communication and control latency times, per 9 measurement rounds. We achieve this by decoding with a scalable FPGA implementation of a Collision Clustering decoder^30^, integrated into Rigetti’s Ankaa^TM^-2 superconducting device’s control system (Fig. 1). We demonstrate decoding times per QEC round faster than the 1 μs threshold for generating measurement data on a superconducting qubit device, sufficient to avoid the backlog problem when the decoder is operated as a streaming decoder, and show logical error suppression for a so-called stability experiment^31^ with up to 25 decoding rounds. Our decoding system’s ability to operate at speeds sufficient to avoid the backlog problem while maintaining low-latency feedback opens the door for experiments involving logical branching, which will be vital for implementing a fault-tolerant universal gate set.

## Results

### Stability experiment on a surface code

In this study, we focus on the stability experiment^31^ implemented with the rotated planar surface code, which is one of the most promising QEC codes due to its high threshold and nearest-neighbour hardware-connectivity requirements^11–13^. The practical implementation of fault-tolerant gates on the surface code has also been extensively studied and developed^16,32–35^. To realize a QEC protocol, Pauli operators called stabilizers are repeatedly measured to detect physical errors. For the rotated surface code, the stabilizer set consists of weight 2 or 4 tensor products of *X* or *Z* operators arranged on a 2D square lattice of qubits. A detector is a product of stabilizer measurements, whose value is deterministic in the case of no errors^36^. For example, we typically assign a detector to the product of consecutive measurements of the same stabilizer. A detector whose value has been flipped from the one expected in the case of no errors is referred to as a defect. Therefore, defects signal the presence of errors. The defect rate at a fixed decoding round is the probability of observing a defect during that round. A syndrome is the set of values of detectors observed during an experiment.

**Fig. 2:**
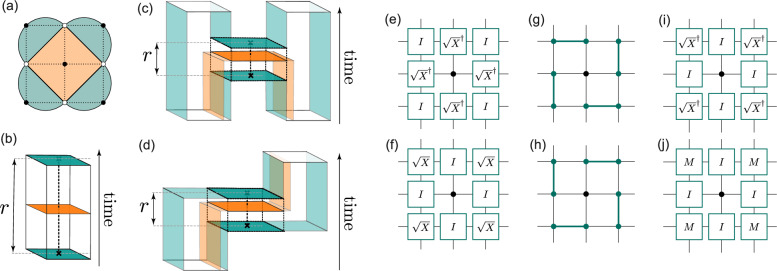
Stability experiment spacetime diagrams and circuit. **a** 2 × 2 stability patch. White dots correspond to data qubits; black dots correspond to ancilla qubits used to measure the stabilizers. Green coloured half-disks are weight-2 *Z*-stabilizers, and the orange square is a weight-4 *X*-stabilizer. The dotted lines indicate the hardware connectivity needed to execute a stability experiment. **b**–**d** Spacetime diagrams for (**b**) a stability experiment, (**c**) lattice surgery, and (**d**) logical patch moving. Orange sheets represent locations where errors can flip the value of a logical observable; green sheets represent places where an error can cause an isolated defect, therefore allowing error strings to terminate; the dashed line shows an example string of undetectable errors that flip the logical observable. The stability experiment is also part of the lattice surgery and logical patch moving routines, as highlighted by the dark sheets in (**c**) and (**d**). To preserve the observable in space, it is measured *r* times. **e**–**j** Gate layers applied during the 8-qubit stability experiment, where the operations in (**e**) prepare the data qubits in the initial state, in (**f**–**i**) map to ancilla qubits the weight-2 *Z*-stabiliser values and in (**j**) measure the weight-2 *Z*-stabiliser values by measuring the ancilla qubits. The qubit placement matches the one shown in Fig. 1(b). Black edges show the qubit hardware connectivity. Edges overlaid with dark green indicate a *CZ* gate. Single-qubit gates are depicted as green boxes with labels indicating the applied gates. Measurement and qubit idling are denoted by *M* and *I*, respectively. The black dot corresponds to the qubit not used in the 8-qubit stability experiment. Gate layer (**e)** is only performed at the beginning of the experiment, while layers (**f**–**j**) are repeatedly executed and correspond to a single syndrome extraction round. Note that ancilla qubits are not reset between rounds. Gate layers (**e**), (**f**) and (**i**) are executed in  ~ 40 ns, (**g**) in  ~ 176 ns, (**h**) in  ~ 112 ns, and (**j**) in ~ 948 ns with an additional 336 ns ring down time. A single QEC round, therefore, takes approximately 1.7 *μ* s.

A stability experiment^31^ tests the capability of a quantum processing unit (QPU) to preserve an observable in space by correctly measuring products of stabilizers (Fig. 2(a,b)). Surface code operations such as lattice surgery^32,37^ (Fig. 2(c)), logical qubit patch movement^16^ (Fig. 2(d)) or the logical Hadamard gate^38,39^ all involve measuring products of stabilizers. In these examples, incorrectly measuring the stabilizer product can introduce a logical error. For instance, in lattice surgery, this stabilizer product determines the logical branching decisions when performing non-Clifford gates, with a logical error meaning the wrong branch is followed. The stability experiment^31^ verifies the ability to protect against these logical errors and in this work, acts as a smaller-scale simulation of logical branching experiments. Such an experiment on a surface code-like patch is performed with an over-complete set of stabilizers whose product is equal to the identity, yet not assigned as a detector. We will refer to this product of stabilizers as a logical observable^36^. In the absence of errors, the logical observable is necessarily measured as  +1. This means that we can verify the ability of a QEC scheme to correctly deduce whether the logical observable has been flipped. We focus on the logical error probability, which, for a fixed number of decoding rounds, is defined as the probability of the decoder incorrectly determining whether the logical observable was flipped during the experiment. The logical observable in a stability experiment is corrupted by stabilizer measurement errors, unlike in a memory experiment, where data errors affect the logical. The stabilizer measurement errors can be suppressed by increasing the number of stabilizer measurement rounds, mirroring how adding more data qubits further protects against data errors in memory experiments. As a result, in stability experiments with sufficiently low physical error rates, the logical error probability is suppressed as the number of decoding rounds increases.

In this work, real-time decoding is demonstrated for a 2 × 2 stability experiment only measuring an overcomplete set of 4 stabilizers of *Z* ⊗ *Z* form, and omitting the single *X* ⊗ *X* ⊗ *X* ⊗ *X* stabilizer that plays no role in decoding. We refer to this experiment as the stability-8 experiment. Omitting the measurement of the middle *X*-stabilizer simplifies the syndrome extraction circuit to be equivalent to that of a circular repetition code and leads to improved logical error rates. In Supplementary Note 7 we also present data decoded offline for a stability-9 experiment on a surface code-like patch that includes the measurement of the middle *X*-stabilizer. Figure 2(e–j) shows the gate layers executed during a stability-8 experiment. Figure 2(e) corresponds to the data qubit preparation layer at the start of the experiment. To obtain a single round of syndrome data, we use the syndrome extraction circuit shown in Fig. 2(f–j), where steps (f–i) map the product of stabilizers to the ancilla qubits, which are then measured in step (j). The measurement outcomes are aggregated to pre-defined detector outcomes, which are inputs to the decoder. The measurement outcomes of the deterministic stabilizers in the first round of syndrome extraction do not form relevant detectors for the stability experiment. This means that for the stability experiment the number of decoding cycles is one fewer than the number of syndrome extraction rounds.

### Integrating an FPGA decoder in the Ankaa-2 control system

We decode with an FPGA decoder integrated into the Ankaa-2 control system (more details on the integration work are given in Methods). Ankaa-2 is a superconducting transmon qubit device, with 84 qubits arranged on a square lattice, and two-qubit gates implemented via tunable couplers^40–42^ (see Methods for details on the Ankaa-2 device). The decoder is an FPGA implementation of a Collision Clustering decoder^30^, which achieves the same logical fidelities as the Union Find algorithm^43^. Modifications were made to the FPGA decoder of Ref. ^30^ to allow for decoding the stability experiment and syndrome extraction circuits without mid-circuit reset operations^44–46^. The FPGA decoder is optimised for speed and scalability to larger systems. On the Ankaa-2 control system, the FPGA decoder is integrated with one of the gate sequencers responsible for gate pulses. In order to simplify the timing requirements in the control system, the decoder is operated at a clock speed of 156.25 MHz, which is the frequency used in the Ankaa-2 control system design (see Methods for more details). The decoder itself has been verified for operating at a clock speed of 400 MHz, allowing for further speed improvements on future control system iterations compared to those demonstrated here. See Ref. ^30^ for a description of the decoding algorithm implemented on the FPGA, as well as a detailed presentation of simulations of the FPGA decoder performance and LUT and register usage when executed at a clock speed of 400 MHz and with various code distances and physical error rates.

The communication between the FPGA decoder and the rest of the control system is performed via custom assembly language-level instructions. We built a prototype compiler that receives a pyQuil^47^ program and adds the necessary instructions to perform real-time decoding and store the results (see Supplementary Note 4 for more details). Our compiler can also merge two PyQuil programs into a logical branching experiment, where the second program is executed conditionally based on a real-time decoded measurement outcome obtained during the first program.

The stabilizer measurement outcomes are automatically passed to the decoder during circuit execution. Once the decoder receives the allocated number of measurements, the measurements are converted to a syndrome, and decoding is performed. The decoded result, along with data storing the decoder speed and status, is written to allocated registers. In a logical branching experiment, the FPGA decoder’s result is communicated in real time to gate sequencers, which then, conditionally on the received result, execute the second program.

### Logical error probability suppression

**Fig. 3:**
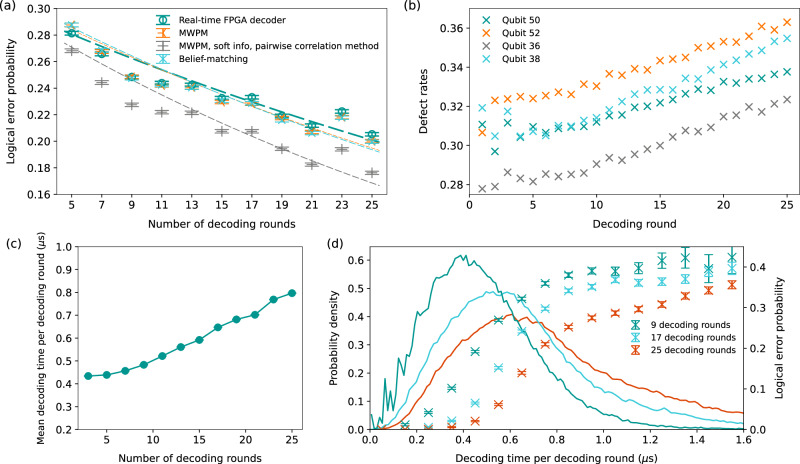
Logical error probabilities and decoder timings. **a** Logical error probabilities for the stability-8 experiment executed on the Ankaa-2 device as a function of number of decoding rounds. We plot logical error probabilities calculated using the decoding results of the real-time FPGA decoder as well as the following offline decoders - minimum-weight perfect matching (MWPM), belief-matching, and a variation of MWPM that uses soft information and the pairwise correlation method to construct the decoding graph. Error bars show the standard error of the mean. Dashed lines are included to guide the eye. Inspecting the fit we see that MWPM and belief-matching slightly outperform the real-time FPGA experiment. **b** Defect rates as a function of decoding round for each of the four ancilla qubits, for a stability-8 experiment performed with 25 decoding rounds. Qubit IDs correspond to the layout shown in Fig. 1(b). **c** Mean decoding time per decoding round for the stability-8 experiment, decoded using the real-time FPGA decoder operating at 156.25 MHz. Error bars are computed as the standard error of the mean and are smaller than the graph point size. **d** Left axis, solid lines: Distributions of decoding times per decoding round for stability-8 experiments performed with 9, 17 and 25 decoding rounds. Right axis, crosses: Logical error probabilities as a function of decoding time. Repetitions of the stability experiment are placed according to their decoding time in bins of width 100 ns, and each logical error probability is estimated as an average within a bin. Error bars are computed as the standard error of the mean.

We perform stability-8 experiments on the Ankaa-2 device for varying numbers of QEC rounds, corresponding to 5 to 25 decoding rounds, and decode in real time. We repeat each experiment 10^5^ times to obtain the probability of a logical error persisting at the end of the QEC routine. In Fig. 3(a), we show that on Ankaa-2, using real-time FPGA decoding the physical error rates are low enough to demonstrate logical error probability suppression with an increasing number of decoding rounds, which is the signature of a successful stability experiment. The logical error probabilities decrease from (28.1 ± 0.1)% at 5 decoding rounds to (20.5 ± 0.1)% at 25 decoding rounds. We also decode offline with minimum-weight perfect matching (MWPM)^48^ and belief-matching^49^ decoders (see Methods for more details) and show in Fig. 3(a) that when using hard measurement data (i.e., the readout signal classified as “0” or “1”), the real-time FPGA decoder results are comparable to these decoders. We also explore decoding with MWPM decoder using soft measurement information (i.e., the in-phase and quadrature components of the raw readout signal)^50^ and a decoding graph constructed with the pairwise correlation method^45,50–53^ (see Supplementary Note 3). This decoder achieves the lowest logical error probability, (17.6 ± 0.1)%, as using soft information and pairwise correlation methods gives the decoder more detailed information, resulting in higher accuracy decoding. In the future, we expect FPGA decoders to be adaptable^54^ so that soft information with the pairwise correlation method could also be used with FPGA decoding.

We observe fluctuations in the logical error probability estimates with varying numbers of decoding rounds, relative to the expected decay; this is likely due to fluctuations in device performance over time, which have been previously shown to occur in superconducting circuit devices, such as fluctuations in the coherence times due to two-level systems (TLSs)^55,56^. Figure 3(b) shows a slight increase in defect rates as further measurement rounds are performed, most likely due to a combination of leakage and heating effects. We believe the limitations on the logical error probability reported here to be largely due to the mid-circuit readout fidelity of the ancilla qubits and the fidelity of the *CZ* gates that were available on the device; the Ankaa-2 processor design was not optimised for these operations in particular. For a more detailed discussion of the device design and relevant error channels, see Methods and Supplementary Note 1.

### Decoding throughput

It is necessary to ensure the decoder has a high enough throughput to avoid the backlog problem^7,21^, which arises when data is generated at a faster rate than it can be decoded. If this is not the case, then, because each conditional operation needs to wait for the ever-growing backlog of data to be decoded before it can be applied, each subsequent conditional operation will be exponentially slower, eventually halting the computation. On the Ankaa-2 device, a single round of syndrome extraction takes ~1.7 μs (see Fig. 2(f–j)). Other superconducting devices have demonstrated a single round of syndrome extraction of approximately 1 μs^17–20^. Figure 3(c) shows that our mean decoding time per round ranges from 0.44 μs when decoding 5 rounds to 0.79 μs when decoding 25 rounds. These values remain below the 1 μs threshold for data generation on a superconducting qubit device, demonstrating that the backlog problem will be avoided when the decoder is operated as a streaming decoder^21–23^. The increase in decoding time per round with more decoding rounds is due to the higher noise in later rounds, leading to increased defect rates in deeper circuits (Fig. 3(b)). As the device improves and the heating and leakage effects are reduced, we expect the mean decoding time per round to grow more slowly as the number of decoding rounds increases. Simulations of the FPGA decoder speed show that the decoding times increase superlinearly with number of decoding rounds, with room for further optimizations to improve the scaling^30^. In the future, we also expect the FPGA decoder to be parallelised, further reducing decoding times.

In Fig. 3(d), we examine the distribution of the decoding times per round (solid lines) and group experiment repetitions in 100 ns bins, based on their decoding time per round, and plot these against the average logical error probability of the experiments in the bins (crosses). We observe that most of our decoding times are well below 1 μs per decoding round. Such experiments correspond to a high-likelihood of correctly decoding the syndrome. A small proportion of experiment repetitions exceed the 1 μs threshold, most likely due to a higher number of defects affecting those experimental runs, which slows down the completion of the decoding. The data represented by crosses indicate that such experiments are associated with worse logical error probabilities.

For practical fault-tolerant computation, we will need lower physical error rates to achieve much lower logical error rates. Lower physical error will lead to lower defect rates and therefore significantly reduce the decoding times. We have seen evidence of this, specifically for our FPGA decoder, in simulations in ref. ^30^. The fast throughput and low latencies of our FPGA decoder at high physical error rates in this work set an upper bound for performance as quantum devices improve and physical error rates decrease. There is also room to more than double the speed of decoding reported in this work, since the FPGA decoder has been verified to be able to operate at 400 MHz, instead of the 156.25 MHz frequency used in these experiments.

### Control and communication latencies

**Fig. 4:**
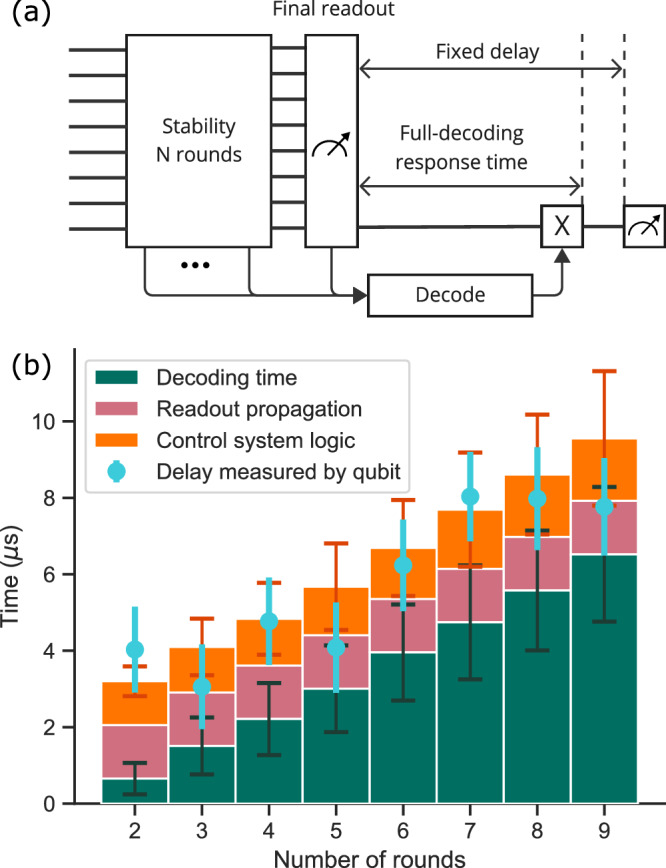
Full decoding response time experiment. **a** The experiment consists of a number of rounds of the stability experiment. After the final readout, the data is sent to the decoder, and a conditional *X* gate is applied to one of the qubits as soon as decoding result is available in the control system. The qubit is measured after some fixed delay. **b** Full decoding response time measurements as a function of number of rounds. Total time measured by the control system FPGA clock cycles (bars) consists of the decoding time measured by decoder onboard metric units (green), waiting for all readouts to reach the sequencer with the decoder (pink), as well as additional logic such as constructing syndrome packets and sending them to the decoder, receiving the result and applying the conditional gate (orange). This is compared to the delay measured from the qubit T1 time (blue points) in order to assess any additional delays in the wiring that might be unaccounted for by the control system (for details see Supplementary Note 5). Error bars on bars are the standard deviation of the decoder times distribution (green) and the total control system time (orange), while the error bars on the blue points are the standard error of the mean of the delay measured by the qubit.

We now focus on the full decoding response time to quantify the contributions of the communication and control latency in our system. We design an experiment that serves as a stepping stone to logical conditional gates. We perform a circuit that conditionally applies a physical gate based on the outcome of decoding the stability-8 experiment (see Fig. 4(a)). After the QEC experiment completes and the decoder has received the allocated number of measurement outcomes of the stability circuit, we start the real-time FPGA decoder and wait for the result. When the result is received, we conditionally apply an *X* gate on the qubit controlled by the gate sequencer on the same FPGA as the decoder. The qubit is read out after a fixed delay, set longer than the worst recorded decoding time (~*N*_rounds_ *μs* for an *N*_rounds_ experiment).

We measure the full decoding response time as a function of the number of QEC rounds (see Fig. 4(b)). Firstly, we record the FPGA clock cycles on the control system sequencer and the cycles on the decoder accelerator. We note that the majority of the full decoding response time can be attributed to the decoding time for a larger number of rounds. At lower physical error rates, the decoding latencies decrease. For example, Ref. ^30^ Table I shows that for *p* = 10^−3^ the FPGA decoder can decode a *d* = 15 surface code memory in 3.75 *μ*s. Therefore, as the device improves to enable larger scale experiments, the decoding latency contribution to the overall response time will significantly decrease. There is also a significant additional latency incurred by the control system delays. In particular, 1.4 *μ*s is the time for the final readout results to reach the decoder, with the remaining 1–1.5 μs (250 − 370 FPGA clock cycles) consisting of additional control system logic: collecting the measurements into packets to be sent to the decoder, sending them via the WISHBONE bus (see Methods), receiving results and performing the conditional logic (see Supplementary Note 4). Furthermore, since the error bars for the timing of the decoder and full control system logic closely match in size, we conclude that the decoding time accounts for the majority of the variance in the full decoding response time.

To check that there are no additional unaccounted delays, we also measure the full decoding response time by using the qubit T1 time as a clock. The measurement distribution after the fixed delay (see Fig. 4(a)) conditional on the final QEC round readout, is a function of the full decoding response time, measurement fidelities, and the qubit’s T1 time (see Supplementary Note 5 for the detailed derivation). Thus, by collecting the relevant reference data immediately prior to running the feedback experiment, we can estimate the delay experienced by the qubit (see Fig. 4(b)) which takes into account any delays that might not be measured by the control system clock. With this, we confirm that there are no additional significant delays as the estimated full decoding response time closely matches the one measured by the control system clock.

For a 9-decoding-round experiment, we find the full decoding response time to be 9.6 μs, including 6.5 μs decoding time and 3.1 μs communication and control latencies. Thanks to the FPGA implementation of our fast decoder and its integration into the Ankaa-2 system, in the small-distance studies reported in this work, we keep the full decoding response time within the order of *d* μs, where *d* is the number of decoding rounds. Maintaining this condition will be crucial when scaling up *d*, as it will ensure that this response time will not be a critical limiting factor for the logical clock speed when implementing non-Clifford operations^21,23^.

## Discussion

Our stability-8 experiment is equivalent to a circular repetition code stability experiment. The repetition code experiment has been previously demonstrated on superconducting qubits in a linear memory configuration, measuring an ability to preserve a logical observable in time^28,53^, whereas we focus on preserving a global invariant across space, leading to different sensitivities to error mechanisms. Since submitting our work, a new study has demonstrated the stability experiment on the heavy-hex code using 23 physical qubits, achieving around 15% logical error probability with 14 syndrome extraction rounds^57^.

Real-time decoding had been previously demonstrated using lookup table decoders on trapped-ion quantum computers^26,27^, superconducting qubits^28^ and a neutral atom device^29^. The memory requirements for lookup table decoders scale exponentially with the number of qubits in the code, so this approach is not feasible for practical fault tolerance, which will require larger codes^4–6,16^.

Recently, the team at Google demonstrated real-time decoding for the distance-5 surface code memory experiment^25^. In their experiment, the measurement data is sent via Ethernet cable from their control system to a software decoder. They demonstrate high throughput, avoiding the backlog problem, by parallelizing decoding^21–23^. The reported decoding response time includes an average of 63 μs to process the final 10 QEC round block. They require an additional 10 μs communication latency to input data to the decoder, and further additional contributions that are not reported, including feedback communication latency. Overall, these numbers are significantly slower than our full decoding response time of 9.6 μs for a 9 decoding rounds experiment. However, we caution against a direct comparison given the significantly different experimental conditions in which our team’s and Google’s experiments were conducted. Our experiment utilises fewer qubits, while operating at a higher noise regime. These conditions have opposite effects on the decoding time, as the decoding time decreases with smaller code sizes but increases with higher noise in the device.

As the code sizes increase, FPGAs have the advantage of having lower inherent latencies and reliable timing. It is challenging to use CPUs for real-time decoding without experiencing rare, but high latency events (see ref. ^25^, Fig. S3). While CPUs can be executed at higher frequencies, FPGAs are more easily parallelizable, such that executing a decoding cycle will require fewer clock cycles than for a CPU decoder. In the long run, the FPGA design can be transferred to ASIC, providing parallelized compute at high clock speeds^30^. Our Collision Clustering decoder FPGA implementation has also been shown to be memory and compute resource efficient, with simulations demonstrating that the decoder uses less than 13 KB of memory for a distance-23 memory experiment^30^.

The speed performance of our FPGA decoder has been extrapolated via simulations to show that, at noise levels just below threshold for memory experiments (*p* = 0.5%), the FPGA decoder will be able to maintain a decoding time below 1 μs per round for memory experiments with distances up to 11^30^. This means that the backlog problem will be avoided when operating the FPGA decoder as a streaming decoder up to large distances. The simulations assume the FPGA decoder operating at 400 MHz frequency, which we expect to be available in future versions of control systems designed to meet real-time decoding requirements. Additionally, in future iterations of the FPGA decoder, we would look to parallelize decoding, which would further improve the throughput.

The fast decoding times of the FPGA decoder, combined with the significant reduction of control and communication latencies achieved by integrating the decoder in the QPU’s control system, suggest that our full decoding time will also remain sufficiently fast to demonstrate larger-scale QEC experiments^3^. We also suggest several strategies for further reducing latency times as the code size is increased. Firstly, readout propagation can be reduced by having a more direct line of communication between readout sequencers and the decoding stack through a standardized interface. To make sure the communication latencies do not become a bottleneck as the experiment size scales, we would look to configure the control system chassis network such that sequencers have as few interchassis “hops” as possible when connecting to the QEC system, using a star topology when possible and appropriate. Secondly, reordering and packing classified measurements before sending them to the decoder can take up to  ~ 400 ns at 9 rounds. In the future, we foresee that such commonly used functionality will have to be offloaded to custom hardware logic attached to the decoder. With these, we do not expect the control system latencies to be a major contributor to the full decoding response time, even as the experiment size increases.

In summary, the experimental results presented show an implementation of a decoding protocol that satisfies the three capabilities required for real-time decoding outlined in Table 1 and provide evidence that the fourth condition can also be satisfied using the FPGA decoder in streaming mode. We demonstrated a logical branching experiment with a scalable FPGA decoder. We showed that integrating the FPGA decoder into the Ankaa-2 control system ensures a low-latency full decoding response time of 9.6 *μ*s with a total of 9 decoding rounds for an 8-qubit experiment. We also report mean decoding times per round under the 1 *μ*s threshold, providing strong evidence that the backlog problem will be avoided when the decoder is operated as a streaming decoder.

We show that the logical error probabilities are suppressed in a stability-8 experiment as the number of decoding rounds increases. However, practical fault tolerance will require lower error rates than those reported here. Fine-tuning the readout pulses to be faster and higher fidelity will be essential to achieving this. While resets are not available, it will also be beneficial to tune the readout pulses to better preserve the classified state of the qubits. Using resets should improve the results^44^ (see exploration of this in Supplementary Note 6). Another primary focus should be to increase the ratio of qubit coherence to two-qubit gate duration, along with reducing leakage and other coherent errors.

In the future, fault-tolerant quantum algorithms that have the potential to outperform classical algorithms will require codes utilizing a large number of operations^3–6,16^. To support this, the FPGA decoder should be updated to be a streaming decoder^21–23^. To improve the accuracy of decoding results, we expect future FPGA implementations to enable updates to the decoding graph in real time^54^, allowing for soft-information, leakage-aware decoding^58^ or correlated decoding^59^. Combining such advances to decoding and improvements to the device with the methods developed in this work will pave the way for logical operations such as magic state teleportation, enabling fault-tolerant quantum algorithms.

## Methods

### Description of control system

The Rigetti control system is built around a configurable card cage chassis. Each physical card in a chassis has inputs or outputs targeting specific RF bands and features high-speed digital-to-analog (DAC) and analog-to-digital (ADC) converters that interface with a Kintex-7 FPGA. The FPGA designs generate (or receive) control signals using one or more sequencers, each of which consists of a dedicated processor, waveform scheduler, and waveform generator. Experiments created in pyQuil are compiled into program binaries, consisting of waveforms and instructions in a proprietary assembly language, which are loaded into each relevant sequencer’s memory by the control system before execution.

The assembly language allows for communication with external modules implemented in the FPGA design via instructions that interact with I/O ports. Additionally, the sequencer assembly language includes instructions that load classified results into a sequencer’s memory so that it can be used by a program.

In order to support experiments that require real-time results, the control system features a low-latency network that distributes each qubit’s most recently classified result when it is published as part of the readout capture pipeline. Onboard-classified data propagates through the control system with a worst case latency time that is calculated beforehand and used when constructing programs to ensure valid results have been received.

### Real-time FPGA decoder integration

When integrating the FPGA decoder and control system, we selected the FPGA design associated with the card for single-qubit gate pulse generation because it had the lowest resource utilization. We used a version of the FPGA decoder featuring a 32-bit WISHBONE interface for communication. We integrated the decoder into the design by adding I/O ports to the gate drive pulse sequencer that initiate READ and WRITE clock cycles over the WISHBONE interface. Via these I/O ports, a sequencer program was able to read from and write to any of the decoder’s addresses in order to perform actions such as writing the syndrome register, starting the decoding process, and reading the decoder result. Signals from other ports on the decoder core, such as the decoder’s status bitfield, were also made available to sequencer programs via an I/O port.

An experiment using an 8-qubit system requires more than one control system chassis. We selected one chassis to be the hub of a star network and connected all other relevant chassis to it, ensuring the lowest achievable results propagation time by limiting the inter-chassis communication to a single hop. Interactions with a decoder were handled by a gate drive sequencer on the hub chassis, which had access to the classified results from the other chassis.

While the sequencer processor clock operates at 250 MHz, we opted to drive the FPGA decoder using the 156.25 MHz clock already present in the design to simplify the timing requirements. We added clock-crossing logic to enable I/O port instructions coming from the sequencer clock domain to result in WISHBONE transactions in the decoder clock domain. Signals received directly from ports on the decoder (e.g., the decoder status bits) were also transitioned to the sequencer clock domain using clock-crossing logic. In the future, we intend to use a single clock domain for the sequencer processor and decoder.

### Ankaa-2 device specifications

The Ankaa-2 device is a superconducting circuit composed of 84 qubits and 149 tunable couplers, all of which are composed of statically capacitively coupled tunable transmons. Each qubit transmon is also coupled to a coplanar waveguide resonator for readout. The device is arranged in a square array with 7 columns and 12 rows of qubits, with a tunable coupler between each neighboring qubit pair, two readout feed lines coupled to each column (in groups of 6), charge drive lines coupled to each individual qubit, and flux lines coupled to the SQUID loop within each individual transmon.

### Software decoding

In addition to decoding in real time with the FPGA decoder, we also obtain decoding results on the same data with offline MWPM^48^ and belief-matching^49^ decoders that use edge weights. We use implementations in PyMatching 2^48^ and BeliefMatching^49^ for the MWPM and belief-matching decoders, respectively. We use Stim^36^ to set up the circuits that represent our experiments and use these circuits to initialise the software decoders. For belief-matching, the belief propagation stage uses the product-sum method with maximum of 5 iterations. Note that in our experiments, we do not observe any improvement when decoding with belief-matching. This is because our decoding graph does not contain hyperedges, as we do not measure the middle stabilizer in the stability-8 experiment. Belief-matching tends to be beneficial when hyperedges are present in the decoding graph.

## Supplementary information

Supplementary Information

Transparent Peer Review file

## Data Availability

The stim circuits and data generated in this study have been deposited in the Zenodo database with the DOI identifier 10.5281/zenodo.13961129.
