# Supplementary material for: Demonstrating real-time and low-latency quantum error correction with superconducting qubits
Source: Nat Commun. 2026 Jun 1;17:7383. doi: 10.1038/s41467-026-73331-6 (PMC13402394; doi:10.1038/s41467-026-73331-6)
Supplement: Supplementary file 1 — Supplementary Information [file 41467_2026_73331_MOESM1_ESM.pdf]

Supplementary Information for "Demonstrating real-time and low-latency quantum error correction with superconducting qubits"

Laura Caune,[1,*] Luka Skoric,[1,†] Nick S. Blunt,[1,‡] Archibald Ruban,[1] Jimmy McDaniel,[2] Joseph A. Valery,[2] Andrew D. Patterson,[3] Alexander V. Gramolin,[4] Joonas Majaniemi,[1] Kenton M. Barnes,[1] Tomasz Bialas,[1] Okan Buğdaycı,[1] Ophelia Crawford,[1] György P. Gehér,[1] Hari Krovi,[4] Elisha Matekole,[4] Canberk Topal,[1] Stefano Poletto,[2] Michael Bryant,[2] Kalan Snyder,[2] Neil I. Gillespie,[1] Glenn Jones,[2] Kauser Johar,[1] Earl T. Campbell,[1,5] and Alexander D. Hill[2]

[1]*Riverlane, Cambridge, CB2 3BZ, UK*
[2]*Rigetti Computing, 775 Heinz Avenue, Berkeley, California 94710, USA*
[3]*Rigetti UK Ltd, 138 Holborn, London, EC1N 2SW, UK*
[4]*Riverlane, Cambridge, Massachusetts 02142, USA*
[5]*Department of Physics and Astronomy, University of Sheffield, UK*

(Dated: April 22, 2026)

Contents

1. Targeted calibration of Ankaa-2 for the stability circuits 1

2. Decoding graphs 2

3. Soft information decoding 3

4. Detailed experiment workflow with real-time decoding and fast-feedback 4

5. Full decoding response time experiment 5

6. Unconditional qubit resets 8

7. Stability-9 experiment results 11

References 12

Supplementary Note 1 – Targeted calibration of Ankaa-2 for the stability circuits

The design of the Ankaa-2 circuit Hamiltonian is optimized towards the application of $iSWAP$ gates. Reconfiguration of the steady state qubit frequencies was required across a sublattice to support the CZ gates used in this work. We compute that the primary source of CZ errors comes from T_ϕ errors, with additional contributions from T_1 errors, higher-state leakage, and swap angle errors (see Supplementary Figure 1(a)). The selected sublattice used in the experiments (qubits 36, 37, 38, 43, 45, 50, 51 and 52) was selected based on the ability to enable the necessary resonances between $|02\rangle$ (or $|20\rangle$) and $|11\rangle$ across the contained qubit pairs, and the performance of the sublattice while in such a configuration. Measurement operations on this chosen sublattice were specifically optimized towards the mid-circuit use case. For example, the readout pulses that were optimised for maximum classification fidelity when positioned at the end of a circuit were observed to cause a large accumulation of leakage to the second-excited state of the repeatedly measured transmons when running the stability circuits over multiple rounds. Reducing the amplitude of readout pulses below an experimentally deduced threshold resulted in a large increase in the probability

* laura.caune@riverlane.com
† luka.skoric@riverlane.com
‡ nick.blunt@riverlane.com

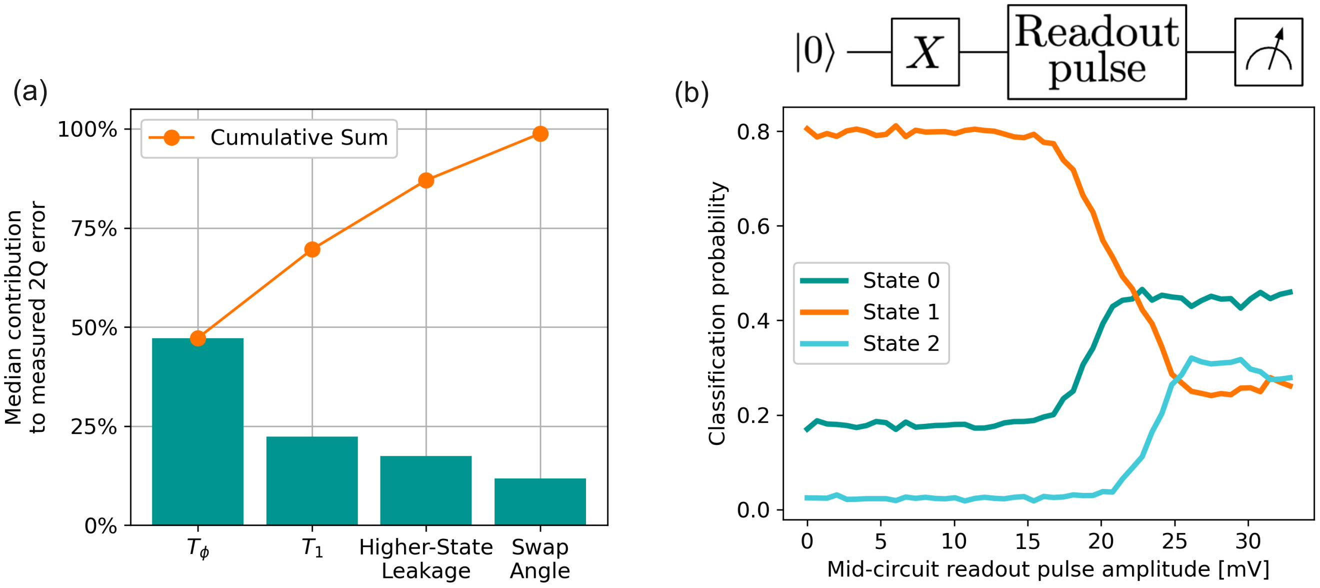

Supplementary Figure 1. **Error budget for CZ gates and measurement-induced leakage experiment.** (a) T_ϕ and T_1 errors are calculated using coherence times measured at both the gate operating point and the qubit idling points (relevant during the short, idle padding delays that play around each gate) [1]. Higher-state leakage error is calculated via the leakage randomized benchmarking protocol [2], using three-state readout to analyze the decay of population in the computational subspace over the course of an interleaved RB experiment. Swap angle error is error attributable to off-target values of θ in the fSIM unitary defined in [3] – for an ideal CZ, $\theta = 0$. Ref. [3] also describes a series of Floquet calibration circuits, the first of which is used here to measure θ for these CZ gates. Considering medians taken over the lattice, T_ϕ contributes almost half of the measured error, with the incoherent error comprised of T_ϕ and T_1 together representing approximately 70%. All together, these four error channels account for a median 99% of measured error for these CZ gates. (b) Representative example of experimental results detailing the dependence of measurement-induced leakage on readout pulse power, shown here for Qubit 52. The circuit consists of an initial preparation of the target qubit in the excited state, before transmission of a test readout pulse, followed by a sufficiently long delay for the resonator to be depopulated before a final measurement with a pulse previously and separately optimized for three-state classification at the end of a circuit. The test readout pulse is the pulse under study, with the process aiming to allow it to be improved for mid-circuit use in stability experiments. The resulting population of the target transmon in each of its three lowest energy states at the end of the circuit is plotted here as a function of the amplitude of the test readout pulse. At low amplitude, population remains largely in the first excited state, as desired, up to a threshold limited by the classifier's three-state confusion matrix. However, there exists an amplitude threshold above which the apparent population rapidly begins to more evenly distribute across the three states. This phenomenon resembles the numerical results presented in [4], which describes the process by which strong resonator drives can suddenly excite coupled transmon transitions to states above their Josephson potential wells. For each ancilla qubit used in this study's stability circuits, these threshold amplitudes were measured and used as upper bounds in further parallel readout optimizations in order to ensure that each mid-circuit measurement would avoid this error mechanism.

that the state of the measured qubit was preserved in the detected state after the readout process (see Supplementary Figure 1(b)). Other considerations included shortening the duration of the readout process by approximately a factor of two compared to standard circuit-end readout, as well as specifically optimizing the ancilla qubit readout pulse parameters for maximum fidelity when performed simultaneously.

Supplementary Note 2 – Decoding graphs

Supplementary Figure 2 shows the decoding graph used by the real-time FPGA decoder and software decoders to decode the stability-8 experiment in this work. The FPGA decoder decoding graph does not use edge weights. For the software decoders that do not use the pairwise correlation method, we use a circuit-level noise model to obtain the edge weights for the decoding graph. Since we did not build a noise model tailored for Ankaa-2, we opted to use a standard noise model used to model the noise in superconducting qubit devices. This model captures the fact that on superconducting qubit devices the two qubit gates and measurements are typically noisier than single qubit

gates. The noise model we use is the same as in Ref. [5], from which we quote the noise channels applied for a fixed probability $p = 0.03$:

- Depolarisation of both qubits after each two-qubit gate with probability p.
- Depolarisation of each idle qubit and after each single-qubit gate, including measurement and reset operations, with probability $p/10$.
- Measurement flip with probability p.

We decided to use $p = 0.03$ based on the median two qubit gate fidelities on Ankaa-2.

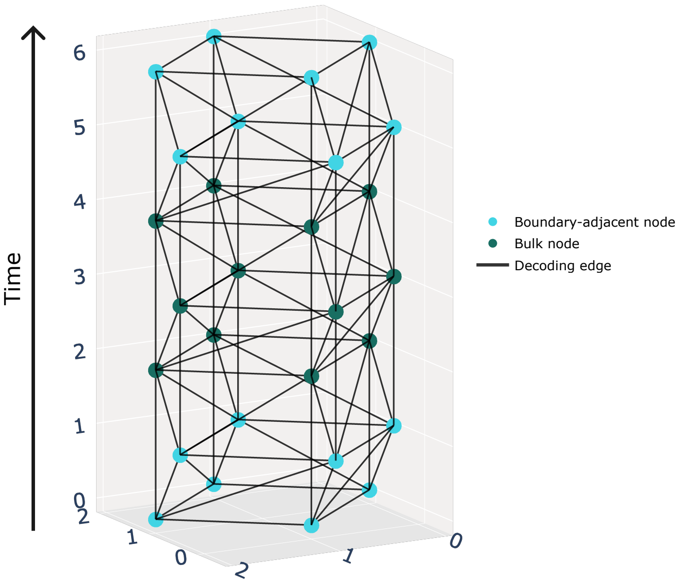

Supplementary Figure 2. **Stability experiment decoding graph.** An example of the decoding graph for 8 rounds of syndrome measurements (equivalent to 7 rounds of detectors). The odd layers are slightly offset so that the edges connecting layers two rounds apart are visible. These edges arise since we are not using mid-circuit resets. The diagonal edges between layers are due to the errors that can happen in the middle of the syndrome extraction circuit and are known as "hook" errors [6]. Boundary-adjacent nodes are detectors that can be matched with a virtual boundary node, and in stability experiments such boundaries are at the top and the bottom rather than on the sides.

Supplementary Note 3 – Soft information decoding

In the the main text we report the lowest logical error probability when using MWPM software decoder that takes a decoding graph from the pairwise correlation method and is dynamically updated with soft measurement information. Here we describe how the soft measurement information is provided to this decoder.

The minimum-weight perfect matching (MWPM) decoder [7] operates on a graph representation of the decoding problem, where detectors correspond to nodes, and possible error mechanisms correspond to edges. The probability of an error mechanism p_e is mapped to the weight of an edge via $w(e) = -\log(p_e/(1-p_e))$, meaning that high-likelihood errors have a low-weight edge in the decoding graph. By finding the lowest-weight perfect matching in the graph, the MWPM algorithm effectively approximates the most probable errors that caused the observed defects. We set the weights in the graph according to the pairwise correlation method [8, 9], which infers the probabilities of error mechanisms based on the defect frequency in the experimental data. To further improve the accuracy of the decoder, we use rich measurement information (soft information) from the qubit readout [10, 11] to dynamically update the probabilities of the measurement error mechanisms in the decoding graph.

Soft information here refers to the integrated voltage $z = (I, Q)$ from the measurement response of the readout resonator, where I and Q are the in-phase and in-quadrature components of the complex signal, respectively. The soft out-

come z is passed to a measurement classifier and converted to an outcome probability $P(\hat{z} \mid z)$ corresponding to the possible measurement outcomes $\hat{z} \in \{0, 1\}$. The hard measurement outcomes are given by $\hat{z} = \text{argmax}[P(0 \mid z), P(1 \mid z)]$.

To train the classifier, we perform a calibration run where we prepare and measure each transmon qubit in the $|0\rangle$- and $|1\rangle$-states 5×10^4 times. We leverage a linear discriminant classifier from the Python library `scikit-learn v1.3.2` [12] as our classifier, and use it to predict the measurement outcome probabilities $P(0 \mid z)$ and $P(1 \mid z)$ for each soft measurement z in the experiment. By preparing an equal number of initial states $|0\rangle$ and $|1\rangle$ for the training data set of the classifier, we ensure the prior probabilities of the two outcomes are $P(0) = P(1) = 1/2$. The outcome probabilities are used to update the edge weights in the decoding graph according to

$$w(z) = -\log\left[\frac{P(z \mid 1-\hat{z})}{P(z \mid \hat{z})}\right], \tag{1}$$

where we use Bayes' theorem to get $P(z \mid \hat{z}) = P(\hat{z} \mid z)P(z)/P(\hat{z})$. We then use the MWPM decoder to decode the hardened syndrome on the updated decoding graph to obtain our logical corrections. The results of this decoder, as discussed in the main text, show a large improvement in the logical fidelity relative to other decoders that use hard measurement data.

Supplementary Note 4 – Detailed experiment workflow with real-time decoding and fast-feedback

The FPGA decoder is integrated into the Ankaa-2 control system. Tasks specific to decoding in real time and with fast feedback are executed by interleaving the qubit operations of the stability-8 experiment with instructions written in the control system's proprietary assembly language, as shown in Supplementary Figure 3. Such tasks include management of the measurement outcomes, communication with the decoder, and carrying out the fast-feedback operation.

The main experiment components are:

1. **Initialise decoder.** Writes to the decoder experiment features (e.g. number of rounds) and decoding configurations (e.g. logical observable definition).

2. **Perform round of gates & measurements.** Executes a single QEC round of the stability-8 experiment. All rounds are identical besides the last in which the data qubits are also measured. Mid-circuit measurements are followed by ring-down delays (included in measurement times), which allow the resonators to decay back to their ground state before the start of the following round [13].

3. **Buffer measurement outcomes.** Stores the outcomes of the latest measurement round in the decoder sequencer's memory. Even though buffering takes less than 0.1 µs, a 1.4 µs delay must take place between measurement and buffering to allow for the measurements to propagate from the readout sequencers to the decoder sequencer. To address this, buffering is swapped with the following round, enabling the readout state to propagate in parallel with each QEC round and preventing the qubits idling mid-circuit. This instruction swapping is applied to all mid-circuit rounds, ensuring that only the last round experiences an actual readout state propagation delay. Note that while Supplementary Figure 3 does not reflect this instruction reordering, the overall duration it shows for a given number of rounds is accurate.

4. **Send measurements to the decoder.** Collects, formats and writes all measurement outcomes to the decoder. This consists of pushing each round's buffer to the sequencer's data stack, combining them into a series of 32-bit binary strings and writing them sequentially to the decoder.

5. **Decode.** The decoder computes the syndrome from measurement outcomes and decodes it using the Collision Clustering algorithm [5]. A write instruction to the decoder initiates decoding, followed by a polling of the decoder's status register, which stalls the program until decoding completes. The decoding result is a Boolean describing whether the decoder computed that the physical errors flipped the stability experiment's logical observable.

6. **Execute fast-feedback operation.** Applies an X gate conditionally on the decoding result and measures the qubit. The X gate is applied if the result is 1, otherwise the qubit is left to idle until measurement for a time equal to the gate's duration.

Supplementary Note 5 – Full decoding response time experiment

As described in the main text, in addition to measuring the response time in the control system, we also measure the delay to applying the conditional operation by considering its effect on the qubit T_1 decay. This experiment allows us to perform full end-to-end response timing purely based on the qubit's physics. In the main text the results of this experiment were acquired on a different day from the data measuring logical error probabilities and throughput

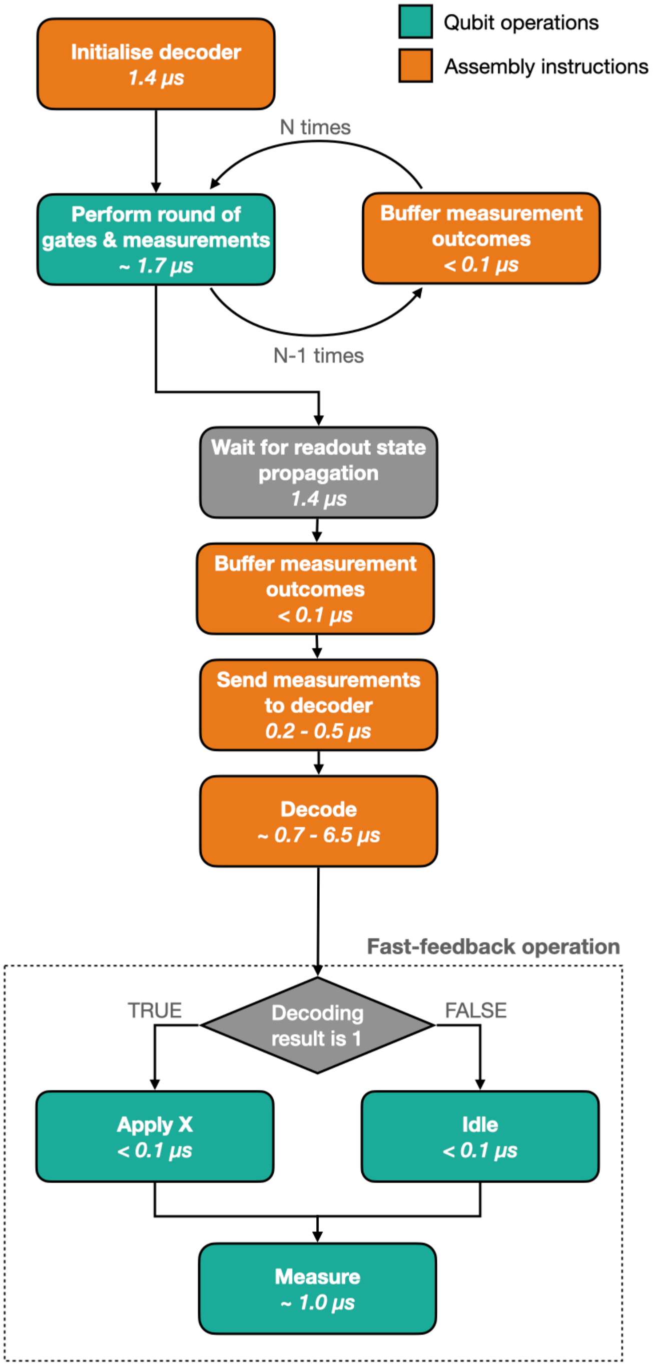

Supplementary Figure 3. **Flowchart for the real-time decoded experiment with fast-feedback.** The execution time for each element is shown in microseconds. When this depends on the number of rounds in the experiment, the times for 2 and 9 measurement rounds are shown instead.

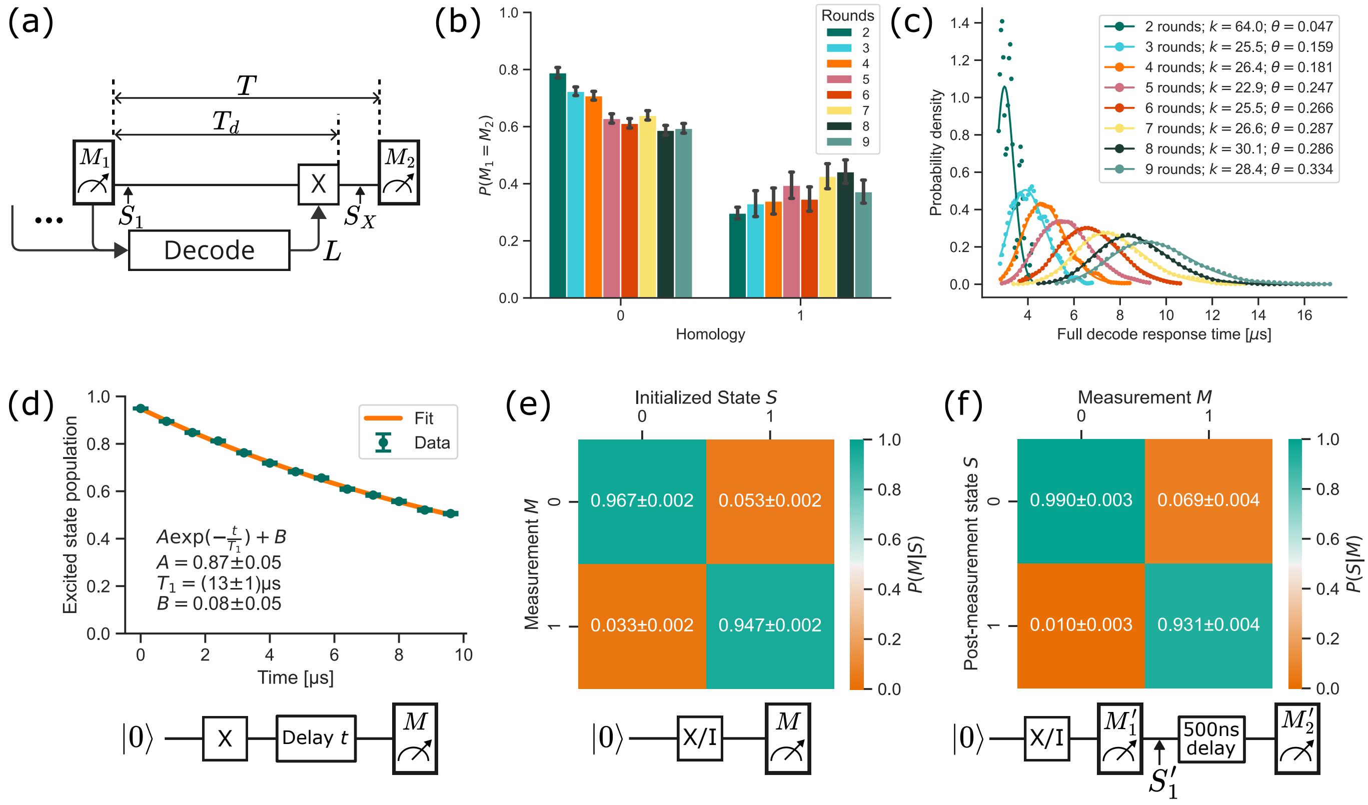

Supplementary Figure 4. **Full decoding response time experiment timing parameters.** (a) The circuit used for timing the full decoding response time. After the final measurement of the stability circuit M_1, the qubit is in state S_1 that is evolving freely while waiting for the conditional gate to be applied. Depending on the logical correction L, an X gate is applied T_d time after M_1, changing the qubit state to S_X. A fixed time T after M_1 a second measurement M_2 is performed. (b) Measured probability of the two measurements matching ($M_1 = M_2$) depending on the computed logical correction L for different number of rounds of the stability experiment. The error bars are standard errors of the mean. (c) Distribution of the full decoding response times as measured by the control system with fits corresponding to the probability density function of Gamma distribution $\Gamma(k, \theta)$ where k, θ are the usual shape and scale parameters respectively. (d–f) Reference data used to compute T_d. The circuits used to acquire the data are shown below the plots. (d) T_1 qubit relaxation time. The data is fitted with the exponential decay curve and error bars are standard deviations of the mean. (e) Measurement confusion matrix $\mathbb{P}(M = i|S = j)\ i, j = 0, 1$. (f) Post-measurement state given the measurement result $\mathbb{P}(S_1' = i|M_2' = j)\ i, j = 0, 1$ acquired by measuring the state twice, with a 500 ns ring-down time between measurements.

leading to a difference in total decoding times due to the variations in device performance and therefore defect rates. The relevant part of the circuit and parameters are shown in Supplementary Figure 4(a).

First, we verify that indeed the conditional operation is applied correctly. We expect that if the decoding result L is $L = 0$ then the first measurement M_1 is equal to the second measurement M_2; and if $L = 1$ then the first measurement M_1 is opposite to the second measurement M_2. We can see that is indeed the case in Supplementary Figure 4(b), although the distribution has significant noise due to multiple microseconds of qubit idling between the measurements.

Second, we derive three reference datasets to classify the qubit's T_1 time decay and measurement characteristics (Supplementary Figure 4(d–f)). All reference data is acquired immediately prior to running the feedback experiment. To simplify the analysis, we assume that state initialisation and single-qubit operations are perfect as their fidelity is much higher than the measurement fidelity. We calculate the qubit's T_1 decay by initializing the $|1\rangle$ state and measuring after a fixed delay t (see Supplementary Figure 4(d)). By fitting an exponential decay, we find

$$\mathbb{P}(M = 1|S = 1, t) = m(t) = A \exp\left(-t/T_1\right) + B \qquad (1)$$
$$A = 0.87 \pm 0.05;\ T_1 = (13 \pm 1)\,\mu\text{s};\ B = 0.08 \pm 0.05.$$

We further evaluate the measurement confusion matrix, which we label $\mathbb{P}(M = i|S = j) = P_{ij}^{M|S}$ by preparing a qubit in state $|S = j\rangle$ and then measuring, obtaining the outcome $M = i$ (see Supplementary Figure 4(e)).

Next, we calculate the distribution of states immediately after the measurement M_1' given the measurement result $\mathbb{P}(S = i|M = j) = Q_{ij}^{S|M}$ by performing a second measurement M_2' after a 500 ns ring down (see Supplementary Figure 4(f)). Here, we use the prime symbol to distinguish these measurements from the ones in Supplementary Figure 4(a). Considering the probability of the second measurement conditioned on the first, the law of total probability and the Markovian property $P(M_2' = i|M_1' = j, S_1' = k) = P(M_2' = i|S_1' = k)$ gives us

$$\begin{aligned}\mathbb{P}(M_2' = i|M_1' = j) &= \sum_{k=0,1} \mathbb{P}(M_2' = i|S_1' = k)\mathbb{P}(S_1' = k|M_1' = j), \\ &= \sum_{k=0,1} P_{ik}^{M|S} Q_{kj}^{S|M},\end{aligned} \tag{2}$$

where S_1' is a state post-M_1'. Since the $|0\rangle$ state is approximately stable and the $|1\rangle$ state decays with a known lifetime, we assume

$$\mathbb{P}(M_2' = 1|S_1' = k) = \begin{cases} P_{10}^{M|S} & \text{if } k = 0, \\ m(t_r) & \text{if } k = 1, \end{cases} \tag{3}$$

with $t_r = 500$ ns being the ring-down delay and $m(\cdot)$ is the T1 decay fit defined in Eq. (1). Using these, Eq. (2) becomes a set of linear equations for $Q_{ij}^{S|M}$ that we solve to acquire the matrix in Supplementary Figure 4(f). All uncertainties are propagated using Python uncertainties package [14].

Having the reference data, we go back to the fast-feedback experiment (Supplementary Figure 4(a)). We consider the probability of measuring $M_2 = 1$ given the decoding result (i.e. logical correction) $\mathbb{P}(M_2 = 1|L = i)$. Using the law of total probability, we expand in state S_1 after the first measurement to give

$$\mathbb{P}(M_2 = 1|L = i) = \mathbb{P}(M_2 = 1|L = i, S_1 = 0)\mathbb{P}(S_1 = 0) + \mathbb{P}(M_2 = 1|L = i, S_1 = 1)\mathbb{P}(S_1 = 1). \tag{4}$$

We can re-express the terms in Eq. (4) in terms of the reference data $P_{ij}^{M|S}, Q_{ij}^{S|M}$ and $m(t)$. The probability to have $S_1 = i$ can be deduced from the M_1 distribution and $Q_{ij}^{S|M}$ as

$$\mathbb{P}(S_1 = i) = \sum_{j=0,1} Q_{ij}^{S|M}\mathbb{P}(M_1 = j). \tag{5}$$

Assuming that the state that starts at $S_1 = 0$ stays at 0 during T_d and gets flipped to 1 by the X gate if $L = 1$, we have

$$\mathbb{P}(M_2 = 1|L = i, S_1 = 0) = \begin{cases} P_{10}^{M|S} & \text{if } L = 0, \\ m(T - T_d) & \text{if } L = 1, \end{cases} \tag{6}$$

where $m(\cdot)$ is the function defined in Eq. (1).

Next, we determine the total time T between the measurements M_1 and M_2 (see Supplementary Figure 4(a)). T consists of the readout propagation delay, control system logic and an additional delay of $\sim N_{\text{rounds}}$ μs inserted on the measurement sequencer at the moment the decoder starts decoding. This can be calculated by timing the program execution on the control system, but here we decide to calculate it directly from the acquired distributions to ensure we are capturing any unaccounted delays. We do this by post-selecting for $L = 0$ cases where there is no conditional operation so the experiment resembles the one in Supplementary Figure 4(f). We have $\mathbb{P}(M_2 = 1|L = 0, S_1 = 1) = m(T)$ and thus, from Eq. (4),

$$\begin{aligned}\mathbb{P}(M_2 = 1|L = 0) &= P_{10}^{M|S}\mathbb{P}(S_1 = 0) + m(T)\mathbb{P}(S_1 = 1), \\ m(T) &= \frac{\mathbb{P}(M_2 = 1|L = 0) - P_{10}^{M|S}\mathbb{P}(S_1 = 0)}{\mathbb{P}(S_1 = 1)},\end{aligned} \tag{7}$$

where we can replace $\mathbb{P}(S_1)$ using Eq. (5) so the right hand is entirely in term of measurable quantities. Taking the inverse of m gives us T. Finally, knowing T, we can compute T_d by considering the distribution in Eq. (4) in the case when the conditional operation is applied ($L = 1$). Using Eq. (6), we have $\mathbb{P}(M_2 = 1|L = 1, S_1 = 0) = m(T - T_d)$, and we use the law of total probability, conditioning on the post-X gate state S_X to obtain

$$\begin{aligned}\mathbb{P}(M_2 = 1|L = 1, S_1 = 1) &= \mathbb{P}(M_2 = 1|S_X = 1)\mathbb{P}(S_X = 1|S_1 = 1, L = 1) + \mathbb{P}(M_2 = 1|S_X = 0)\mathbb{P}(S_X = 0|S_1 = 1, L = 1) \\ &= m(T - T_d)\left(1 - e^{-T_d/T_1}\right) + P_{10}^{M|S} e^{-T_d/T_1}.\end{aligned} \tag{8}$$

Note that $m(T - T_d) = [m(T) - B]e^{T_d/T_1} + B$, so setting $\alpha_d = e^{-T_d/T_1}$ and putting everything together in Eq. (4), we find

$$\mathbb{P}(M_2 = 1|L = 1) = \left(\frac{m(T) - B}{\alpha_d} + B\right)\mathbb{P}(S_1 = 0) + \left[\left(\frac{m(T) - B}{\alpha_d} + B\right)(1 - \alpha_d) + P_{10}^{M|S}\alpha_d\right]\mathbb{P}(S_1 = 1), \tag{9}$$

which is a quadratic equation for α_d in terms of measurable quantities and quantities derived in Eqs. (1), (5) and (7). Note that the quantity evaluated by putting in the experimental data is $\langle\alpha_d\rangle = \mathbb{E}\left(e^{-T_d/T_1}\right)$ where T_d is a random variable. Computing $\tilde{T_d} = -T_1 \log \mathbb{E}(e^{-T_d/T_1})$ gives us a biased estimate for $\mathbb{E}(T_d)$. In the following, we show that this bias is negligible given the distribution of decoding times, allowing us to estimate $\mathbb{E}(T_d) \approx -T_1 \log\langle\alpha_d\rangle$.

We model T_d as distributed by the Gamma distribution $T_d \sim \Gamma(k, \theta)$ where k, θ are the shape and scale parameters respectively. The probability density function is defined as:

$$f(x; k, \theta) = \frac{x^{k-1}e^{-x/\theta}}{\theta^k\Gamma(k)}. \tag{10}$$

In Supplementary Figure 4(c) we see that this distribution fits well the full decoding response times as measured by the control system clock, and we have no reason that this would stop being true for the full response time as experienced by the qubit. By the law of the unconscious statistician (LOTUS):

$$\begin{aligned}\mathbb{E}\left(e^{-T_d/T_1}\right) &= \int_0^\infty e^{-x/T_1}\frac{x^{k-1}e^{-x/\theta}}{\theta^k\Gamma(k)}dx \\ &= \frac{1}{\theta^k\left(1/\theta + 1/T_1\right)^k}\int_0^\infty \left(\frac{1}{T_1} + \frac{1}{\theta}\right)^k \frac{x^{k-1}e^{-x\left(\frac{1}{T_1}+\frac{1}{\theta}\right)}}{\Gamma(k)}dx \qquad (11)\\ &= \left(1 + \frac{\theta}{T_1}\right)^{-k}, \qquad (12)\end{aligned}$$

where the integral in Eq. (11) is 1 as it is integrating the probability density function of a Gamma distribution $\Gamma\left(k, (1/T_1 + 1/\theta)^{-1}\right)$ across the full domain. Hence, using Eq. (12):

$$\tilde{T_d} = -T_1 \log \mathbb{E}\left(e^{-T_d/T_1}\right) = T_1 k \log\left(1 + \frac{\theta}{T_1}\right). \tag{13}$$

If $\theta \ll T_1$, we can expand the logarithm to first order in Taylor series and use the expected value of the Gamma distribution $\mathbb{E}(T_d) = k\theta$ to find:

$$\tilde{T_d} \approx T_1 k\left[\frac{\theta}{T_1} + \mathcal{O}\left(\frac{\theta^2}{T_1^2}\right)\right] = \mathbb{E}(T_d)\left[1 + \mathcal{O}\left(\frac{\theta}{T_1}\right)\right]. \tag{14}$$

As we can see from Supplementary Figure 4(c), the scale parameter for the range of rounds used in this experiment is $\theta < 0.4$. With this, $\theta/T_1 \lesssim 3\%$ is a negligible bias in the estimator as the relative standard error is greater than 10%. Even if there are additional delays unaccounted for in the distributions in Supplementary Figure 4(c), we do not expect these to change θ significantly enough to invalidate this analysis.

Supplementary Note 6 – Unconditional qubit resets

While the experiments described in the main text did not involve resetting ancilla qubits between QEC rounds, we have also explored and describe here a specific implementation of unconditional qubit resets. Note that these resets were implemented during the final stage of the project and we did not evaluate their performance with the real-time FPGA decoder. Here we present evidence of the improved logical error rates when using unconditional resets with a software decoder.

Recall that qubits can be reset to their ground state either by passively waiting (to achieve a thermal equilibrium with the cold bath, which happens on a time scale much longer than the relaxation time T_1) or by actively applying additional control pulses, which may or may not be conditional on the qubit state. Conditional (or measurement-based) resets work by first measuring the qubit and then applying an X gate if the measurement outcome is “1”.

In contrast, unconditional resets apply the same pulse sequence regardless of the qubit's state. While conditional resets cannot improve the performance of QEC circuits, fast and high-fidelity unconditional resets offer a significant advantage by doubling the number of measurement errors that can be tolerated by a stability experiment [15].

Unconditional resets work by transferring the excited-state population of the qubit to a lossy environment, such as the qubit's readout resonator [16, 17]. Although the transferring rate is limited by the qubit-resonator coupling strength g, the resonator can be quickly thermalized with the environment if it is strongly coupled to the cold bath: $1/\kappa \ll T_1$, where κ is the dissipation rate of the resonator. This requirement is well satisfied for the Ankaa-2 qubits, where the typical values are $T_1 \approx 15$ μs and $\kappa/2\pi \approx 3$ MHz.

In this work, we investigated the double-drive reset of population (DDROP) protocol [16], which is particularly well suited to the Ankaa-2 control system. Compared to the parametric reset protocol of Ref. [17], the DDROP scheme requires much smaller instantaneous bandwidth of the control electronics. This is because both the qubit and the resonator drives used for DDROP are detuned from their respective resonant frequencies by only several dozen MHz. The DDROP protocol is also applicable to fixed-frequency qubits, which is another advantage over the parametric resets requiring flux-tunable qubits.

Let us now explain how the DDROP protocol works. It exploits the qubit-resonator coupling, which is described by the well-known Jaynes–Cummings model [18]. Transmon qubits typically operate in the dispersive regime, when the qubit-resonator coupling is weak compared to their detuning: $g \ll |\Delta|$, where $\Delta \equiv \omega_q - \omega_r$ and ω_q/ω_r is the bare frequency of the qubit/resonator. In this regime, the Jaynes–Cummings Hamiltonian can be written as

$$\begin{aligned} \hat{H} &= \hbar\left(\omega_r + \chi\hat{\sigma}_z\right)\hat{a}^\dagger\hat{a} + \frac{\hbar}{2}\left(\omega_q + \chi\right)\hat{\sigma}_z \\ &= \hbar\omega_r\hat{a}^\dagger\hat{a} + \frac{\hbar}{2}\left(\omega_q + \chi + 2\chi\hat{a}^\dagger\hat{a}\right)\hat{\sigma}_z, \end{aligned} \tag{1}$$

where $\hbar$ is Planck's constant, $\hat{\sigma}_z$ is the Pauli Z operator of the qubit, $\hat{a}^\dagger$ and $\hat{a}$ are, respectively, the creation and the annihilation operators of the resonator (which is modeled as a harmonic oscillator), and $\chi = g^2/\Delta$ is the so-called dispersive shift (in our case, $\chi < 0$ since $\Delta < 0$).

Two effects caused by the qubit-resonator interaction are immediately apparent from this Hamiltonian. As follows from the first line of Eq. (1), the resonator frequency ω_r acquires a shift of $\pm\chi$, whose sign depends on the qubit's state (i.e., the ± 1 eigenvalue of the $\hat{\sigma}_z$ operator). This effect is widely used for performing a dispersive readout of the qubit by probing the frequency of its readout resonator [18]. The second line in Eq. (1) implies that the qubit frequency ω_q also experiences two frequency shifts: a constant shift χ (called the Lamb shift) and a variable shift $2\chi n$ (known as the ac-Stark shift), whose size depends on the number of photons, $n = \langle\hat{a}^\dagger\hat{a}\rangle$, populating the resonator.

In the dispersive regime, driving the readout resonator at a frequency $\omega_r + \delta_r$ (we will refer to δ_r as the resonator detuning) leads to a steady state with the average number

$$\bar{n}_{g,e}(\delta_r) \approx \frac{n_0}{1 + 4\left(\delta_r \pm \chi\right)^2/\kappa^2} \tag{2}$$

of photons in the resonator, where n_0 is a constant proportional to the drive's power [19]. This equation assumes that the qubit is in a specific state: either ground, $|g\rangle$, or excited, $|e\rangle$. For each value of the resonator detuning, the photon number (2) takes two values: $\bar{n}_g$ if the qubit is in $|g\rangle$ (which corresponds to the "+" sign in the denominator) and $\bar{n}_e$ if the qubit is in $|e\rangle$ (the "−" sign in the denominator). Equation (2) describes two Lorentzian peaks, which are centered at $\pm\chi$ and have the amplitude n_0 and the full width at half maximum (FWHM) κ.

Due to the Stark effect, the resonator being populated with $\bar{n}_{g,e}$ photons causes the qubit frequency to shift by

$$\delta_q \approx 2\chi\bar{n}_{g,e}(\delta_r) \tag{3}$$

relative to its bare value ω_q (we call δ_q the qubit detuning). Therefore, we expect that if we drive the resonator at different frequencies and simultaneously probe the qubit's transition frequency, the latter would be shifted by δ_q and the observed dependence of δ_q on δ_r would approximately follow Eqs. (2) and (3). This is indeed the case for Ankaa-2 qubits, as illustrated by Supplementary Figure 5(a) showing the results of two-tone spectroscopy for Qubit 76.

The two-tone spectroscopy was performed in the following way [19]. We first prepared an equal superposition of the $|g\rangle$ and $|e\rangle$ states by applying an $R_x(\pi/2)$ rotation to the qubit initialized in the ground state. We then simultaneously drove the qubit and its readout resonator for 1.5 μs while keeping the amplitudes of the drives fixed and varying their frequencies. After we turned off the drives, we waited for 360 ns to let the resonator relax to its ground state. Finally, we measured the qubit state using a regular Ankaa-2 readout pulse. The measurement outcomes averaged over 200 shots are shown in Supplementary Figure 5(a) by color, with red/blue corresponding to the ground/excited state. Two overlapping Lorentzian dips can be clearly seen in Supplementary Figure 5(a). Note that the Lorentzians are

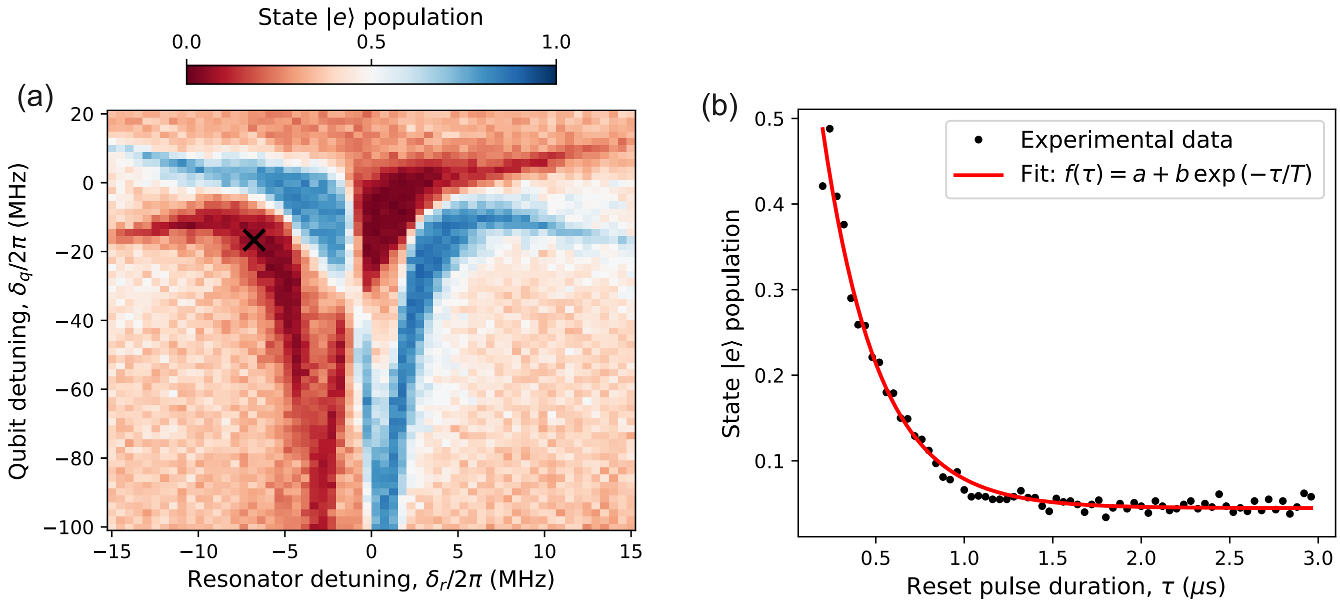

Supplementary Figure 5. **Calibration of DDROP resets for Qubit 76 of Ankaa-2.** (a) Results of two-tone spectroscopy. The dependence of the qubit detuning δ_q on the resonator detuning δ_r approximately follows Eq. (3). The color represents the measured population of the qubit's state $|e\rangle$ averaged over 200 shots. Two distorted Lorentzian dips can be clearly seen: the left one corresponds to the photon number $\bar{n}_e$ (red color), while the right one corresponds to $\bar{n}_g$ (blue color). The separation between the two dips is about $2\chi/(2\pi) \approx -5$ MHz and the FWHM of each Lorentzian is $\kappa/(2\pi) \approx 3$ MHz. The black cross indicates the combination of drive detunings used for implementing the unconditional reset pulse of panel (b). (b) Excited-state population as a function of the reset pulse duration. Experimental data averaged over 1000 shots are shown with black markers. The red curve is a fit to the data with a function $f(\tau) = a + b\exp(-\tau/T)$. We obtained the following best-fit parameters: steady-state population $a = 0.045$ and decay constant $T = 0.31$ μs.

distorted due to a non-linear dependence of the dispersive shift on $\bar{n}_{g,e}$ that we have neglected [19]. We performed all our measurements using Rigetti's Quantum Cloud Services (QCS) platform, which allows for pulse-level control.

The DDROP protocol [16] is based on the same principles as the two-tone spectroscopy we just described. To reset a qubit, we apply two simultaneous drives at the frequencies corresponding to the left Lorentzian dip in Supplementary Figure 5(a). After applying the two drives for sufficiently long time (of about $10/\kappa$), the coupled qubit-resonator system is driven to the steady state $|g, \bar{n}_g\rangle$. Then the drives are turned off, and the system spontaneously relaxes to the target state $|g, 0\rangle$, which happens on a time scale of about $1/\kappa$. Note that the right dip in Supplementary Figure 5(a) (shown in blue) corresponds to the steady state $|e, \bar{n}_e\rangle$. Therefore, by choosing an appropriate combination of drive frequencies, this protocol can also be used for bringing the qubit to the $|e\rangle$ state instead of $|g\rangle$.

We used the following procedure to coarsely calibrate unconditional resets for Ankaa-2. We kept the resonator drive amplitude fixed at the level used for mid-circuit measurements, which in turn was optimized to avoid leakage (as described in Methods). We then fixed the qubit drive strength at some initial value and performed two-tone spectroscopy. Based on the two-tone spectroscopy results, we chose a combination of the drive frequencies corresponding to the left Lorentzian in Fig. 5(a) (one of the possible choices is shown by the black cross in the figure). Having fixed the amplitude of the resonator drive and the frequencies of both drives, we then performed a sweep of the qubit drive amplitude to minimize the excited-state population measured after the reset pulse. Finally, we tested the performance of the DDROP resets by varying the duration of the simultaneous drives and measuring the resulting state $|e\rangle$ population. The corresponding experimental results are shown in Fig. 5(b) and are also fitted with an exponentially decaying function. For Qubit 76 shown in Fig. 5(b), we extracted the steady-state population of 0.045 and the decay time constant of 0.31 μs, but similar values were obtained for other qubits. Based on these results, we fixed the reset pulse duration at 1.5 μs. In our experience, this time can be reduced by increasing the resonator drive strength, but this can also increase leakage (see Methods). Note that the observed steady-state population is likely limited by the measurement fidelity rather than by the performance of the DDROP resets.

Figure 6 shows the logical error probability for the stability-8 experiment performed both with and without resetting ancillas between syndrome extraction rounds. We decoded offline with the MWPM decoder using soft information and constructing the decoding graph with the pairwise correlation method. Our results indicate that resetting the ancilla qubits improves the logical error probability. We plan to further investigate the DDROP protocol elsewhere,

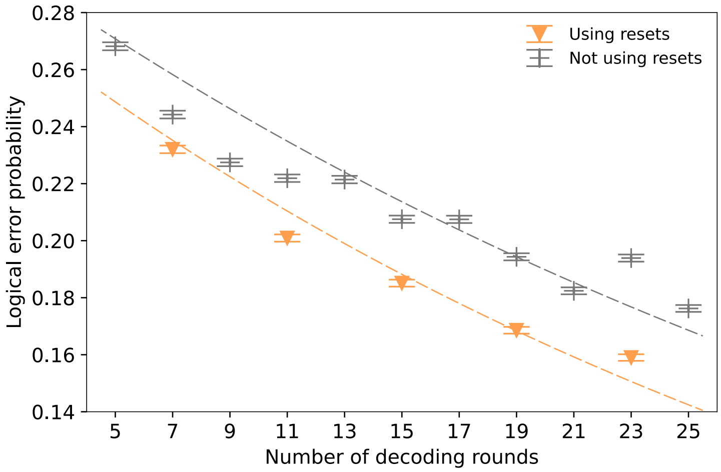

Supplementary Figure 6. **Impact of unconditional resets on the logical error probability.** Shown is the logical error probability as a function of the number of decoding rounds for stability-8 experiments with and without using unconditional resets after each syndrome extraction round. The syndrome data are decoded with a software implementation of the MWPM decoder using soft information and the pairwise correlation method to construct a decoding graph. Error bars show the standard error of the mean. Dashed lines are included to guide the eye.

including the possibility to adapt it for resetting transmons in higher excited states.

Supplementary Note 7 – Stability-9 experiment results

The stability-8 experiment results presented in the main text measure only the Z-stabilizers. However, implementing fault-tolerant quantum computation on the surface code requires measuring both X and Z stabilizers. Here we present results of stability experiment on 9 physical qubits (stability-9) that measures both the weight-2 Z stabilizers as well as the middle, weight-4 X stabilizer. The stability-9 experiments were not decoded in real-time, instead here the data was decoded offline using MWPM. Supplementary Figure 7(a) shows that for the stability-9 experiment logical error probabilities decrease from $(40.1 \pm 0.2)\%$ at 5 decoding rounds to $(38.82 \pm 0.2)\%$ at 27 decoding rounds. The logical error probabilities for the stability-9 experiment are higher than for the stability-8 experiment, as measuring the weight-4 X stabilizer adds additional operations to the circuit, which in turn increases the defect rates of the experiment as shown in Supplementary Figure 7(b). All data for the experiment performed, including Stim [20] circuits, measurement results, logical error probabilities and defect rates, can be found along with the other supporting data available in Zenodo (DOI identifier https://doi.org/10.5281/zenodo.13961129).

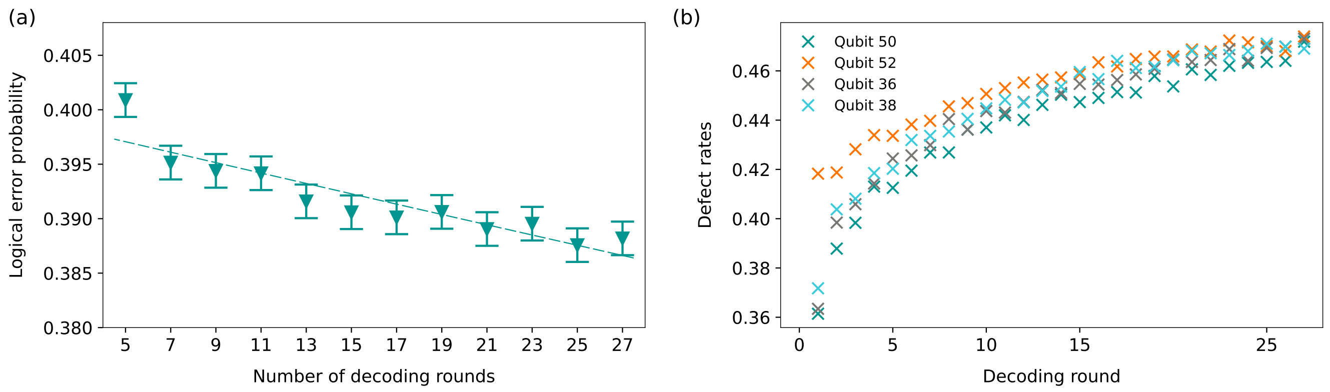

Supplementary Figure 7. **Logical error probabilities and defect rates for the stability-9 experiment.** (a) Logical error probabilities for the stability-9 experiment with 27 decoding rounds executed on the Ankaa-2 device as a function of number of decoding rounds. The syndrome data is decoded offline with the MWPM decoder. The experiment was run with 27 decoding rounds and the results with fewer decoding rounds were obtained by decoding subsets of data with the corresponding number of rounds. Error bars show the standard error of the mean. Dashed lines are included to guide the eye. (b) Defect rates as a function of decoding round for each of the four ancilla qubits measuring the Z stabilizers for a stability-9 experiment. Qubit IDs correspond to the layout shown in the main text. Note that the measurement outcomes of the middle X stabilizer are not used for decoding and therefore the middle ancilla, corresponding to qubit 44, does not have an associated defect rate.

Supplementary References

---

[1] E. A. Sete, V. Tripathi, J. A. Valery, D. Lidar, and J. Y. Mutus, Physical Review Applied **22**, 014059 (2024).
[2] C. J. Wood and J. M. Gambetta, Phys. Rev. A **97**, 032306 (2018).
[3] F. Arute, K. Arya, R. Babbush, D. Bacon, J. C. Bardin, R. Barends, A. Bengtsson, S. Boixo, M. Broughton, B. B. Buckley, D. A. Buell, B. Burkett, N. Bushnell, Y. Chen, Z. Chen, Y.-A. Chen, B. Chiaro, R. Collins, S. J. Cotton, W. Courtney, S. Demura, A. Derk, A. Dunsworth, D. Eppens, T. Eckl, C. Erickson, E. Farhi, A. Fowler, B. Foxen, C. Gidney, M. Giustina, R. Graff, J. A. Gross, S. Habegger, M. P. Harrigan, A. Ho, S. Hong, T. Huang, W. Huggins, L. B. Ioffe, S. V. Isakov, E. Jeffrey, Z. Jiang, C. Jones, D. Kafri, K. Kechedzhi, J. Kelly, S. Kim, P. V. Klimov, A. N. Korotkov, F. Kostritsa, D. Landhuis, P. Laptev, M. Lindmark, E. Lucero, M. Marthaler, O. Martin, J. M. Martinis, A. Marusczyk, S. McArdle, J. R. McClean, T. McCourt, M. McEwen, A. Megrant, C. Mejuto-Zaera, X. Mi, M. Mohseni, W. Mruczkiewicz, J. Mutus, O. Naaman, M. Neeley, C. Neill, H. Neven, M. Newman, M. Y. Niu, T. E. O'Brien, E. Ostby, B. Pató, A. Petukhov, H. Putterman, C. Quintana, J.-M. Reiner, P. Roushan, N. C. Rubin, D. Sank, K. J. Satzinger, V. Smelyanskiy, D. Strain, K. J. Sung, P. Schmitteckert, M. Szalay, N. M. Tubman, A. Vainsencher, T. White, N. Vogt, Z. J. Yao, P. Yeh, A. Zalcman, and S. Zanker, Observation of separated dynamics of charge and spin in the fermi-hubbard model (2020), arXiv:2010.07965 [quant-ph].
[4] R. Shillito, A. Petrescu, J. Cohen, J. Beall, M. Hauru, M. Ganahl, A. G. Lewis, G. Vidal, and A. Blais, Phys. Rev. Appl. **18**, 034031 (2022).
[5] B. Barber, K. M. Barnes, T. Bialas, O. Buğdaycı, E. T. Campbell, N. I. Gillespie, K. Johar, R. Rajan, A. W. Richardson, L. Skoric, C. Topal, M. L. Turner, and A. B. Ziad, A real-time, scalable, fast and resource-efficient decoder for a quantum computer, publisher: Nature Publishing Group.
[6] E. Dennis, A. Kitaev, A. Landahl, and J. Preskill, Topological quantum memory.
[7] O. Higgott and C. Gidney, Quantum **9**, 1600 (2025).
[8] S. T. Spitz, B. Tarasinski, C. W. Beenakker, and T. E. O'Brien, Advanced Quantum Technologies **1**, 1800012 (2018).
[9] E. H. Chen, T. J. Yoder, Y. Kim, N. Sundaresan, S. Srinivasan, M. Li, A. D. Córcoles, A. W. Cross, and M. Takita, Physical Review Letters **128**, 110504 (2022).
[10] C. A. Pattison, M. E. Beverland, M. P. da Silva, and N. Delfosse, arXiv preprint arXiv:2107.13589 (2021).
[11] H. Ali, J. Marques, O. Crawford, J. Majaniemi, M. Serra-Peralta, D. Byfield, B. Varbanov, B. M. Terhal, L. DiCarlo, and E. T. Campbell, Physical Review Applied **22**, 044031 (2024).
[12] F. Pedregosa, G. Varoquaux, A. Gramfort, V. Michel, B. Thirion, O. Grisel, M. Blondel, P. Prettenhofer, R. Weiss, V. Dubourg, J. Vanderplas, A. Passos, D. Cournapeau, M. Brucher, M. Perrot, and E. Duchesnay, Journal of Machine Learning Research **12**, 2825 (2011).
[13] A. Bengtsson, A. Opremcak, M. Khezri, D. Sank, A. Bourassa, K. J. Satzinger, S. Hong, C. Erickson, B. J. Lester, K. C. Miao, A. N. Korotkov, J. Kelly, Z. Chen, and P. V. Klimov, Physical Review Letters **132**, https://doi.org/10.1103/physrevlett.132.100603 (2024).

[14] E. O. Lebigot, Uncertainties: a python package for calculations with uncertainties.
[15] G. P. Gehér, M. Jastrzebski, E. T. Campbell, and O. Crawford, To reset, or not to reset – that is the question (2024), arXiv:2408.00758 [quant-ph].
[16] K. Geerlings, Z. Leghtas, I. M. Pop, S. Shankar, L. Frunzio, R. J. Schoelkopf, M. Mirrahimi, and M. H. Devoret, Phys. Rev. Lett. **110**, 120501 (2013).
[17] Y. Zhou, Z. Zhang, Z. Yin, *et al.*, Nature Communications **12**, 5924 (2021).
[18] P. Krantz, M. Kjaergaard, F. Yan, T. P. Orlando, S. Gustavsson, and W. D. Oliver, Appl. Phys. Rev. **6**, 021318 (2019).
[19] L. Tosi, I. Lobato, M. F. Goffman, C. Metzger, C. Urbina, and H. Pothier, Phys. Rev. Research **6**, 023299 (2024).
[20] C. Gidney, Quantum **5**, 497 (2021).
